# M. Bernard on the Absorption of Aliments

**Published:** 1851-04

**Authors:** 


*M. Bernard on the Absorption of Aliments.*—The following is the substance of a memoir recently presented to the Academy of Sciences, by M. Bernard, on the absorption of alimentary substances by the chyle vessels.

All alimentary substances are reduced in the intestinal canal to three principal matters, viz., saccharine, albuminous, and fatty emulsion.

*Absorption of Sugar by the Chyle Vessels.*—On administering large quantities of cane sugar to the mammalia, M. Bernard has always found that substance in the blood of the vena portæ, whereas, the thoracic duct contains no trace of that substance. The author hence concludes, that sugar is exclusively absorbed from the digestive canal by the system of the vena portæ, and that saccharine substances traverse the liver before they reach the lungs.

In thus traversing the liver, saccharine substances undergo an important transformation. If a solution of two or three scruples of cane sugar be injected into the superficial veins of a dog, this substance, instead of being assimilated, is excreted with the urine in a few minutes. But, if the sugar be thrown into one of the branches of the vena portæ, and thus forced to traverse the liver, it is no longer eliminated, but assimilated and combined with the blood, just as occurs after normal digestion We may conclude from this that absorption by the system of the vena portæ is a necessary condition for the assimilation of sugar.

*Absorption of Albumen.*—When a weak solution of albumen from the egg is thrown into the jugular vein of a dog or rabbit, the urine becomes albuminous some time afterwards. M. Bernard considers that this shows a difference between the albumen of the egg and the albumen of the blood; and that the former requires to be modified before it can become incorporated with the living body. The modification takes place in the liver; for, when the albumen from an egg is injected into the vena portæ, it remains in the blood and is not eliminated by the kidneys. These experiments tend to prove that albuminous matters are absorbed exclusively by the vena portæ; they likewise show that cane sugar is subject to the same law, and that both can only be assimilated by undergoing the influence of the liver.

*Absorption of Fat.*—In a former memoir, M. Bernard demonstrated that fatty matters require to be converted into an emulsion by the pancreatic juice before they can be absorbed in the stomach. We might hence, *à priori*, conclude, that fatty matters do not require to pass through the liver, and this is the case. Still, however, it is certain, that fatty matters are sometimes obsorbed by the branches of the vena portæ as well as by the chyle vessels.—*Ibid from Bulletin de l' Acad.*

